# Supplementary figures and images for: ABCG5 and ABCG8 Are Involved in Vitamin K Transport
Source: Nutrients. 2023 Feb 16;15(4):998. doi: 10.3390/nu15040998 (PMC9966996; doi:10.3390/nu15040998)

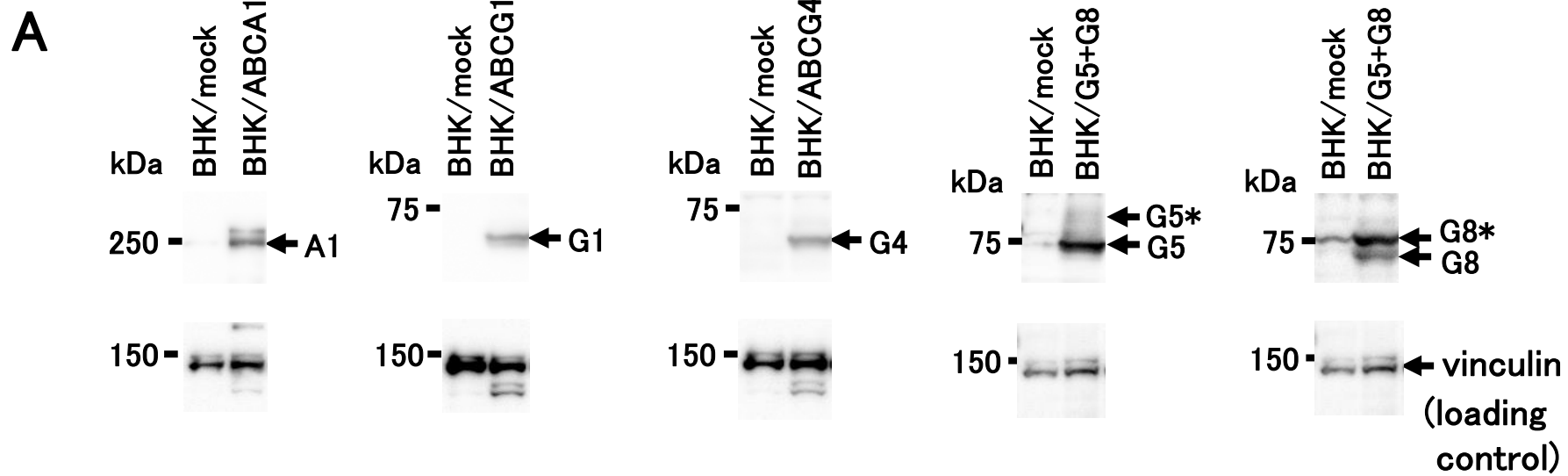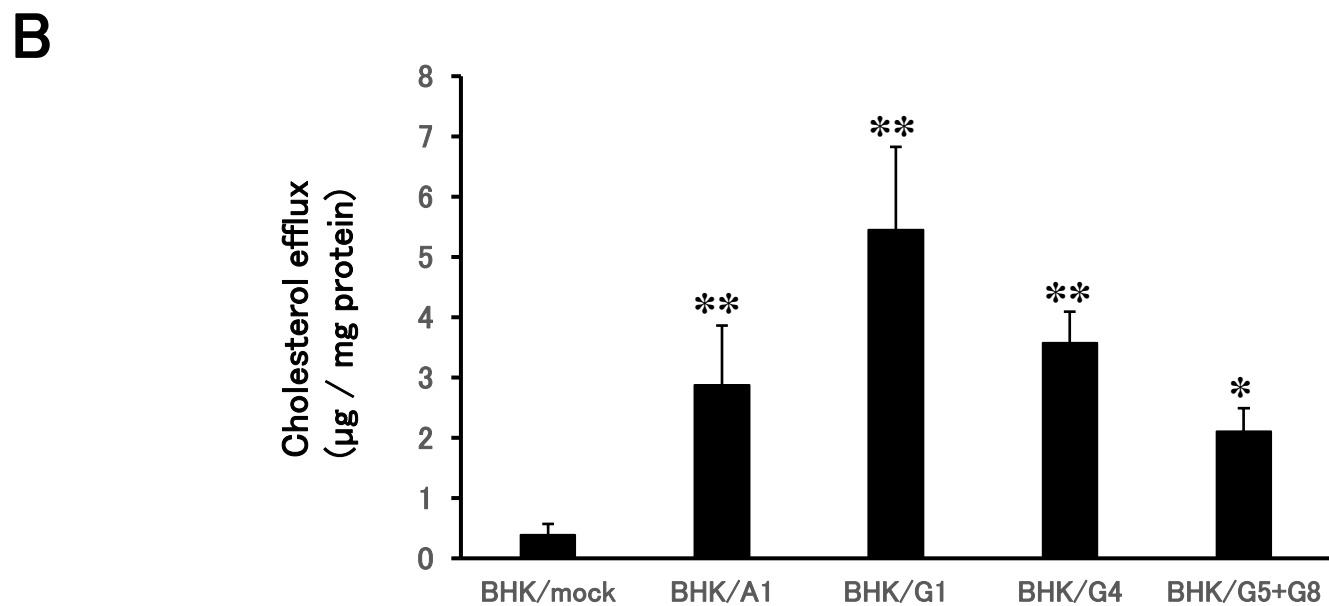

Supple Fig. 1

Supplement: Supplementary file 1 [file nutrients-15-00998-s001.zip › nutrients-2225101-supplementary.pdf]
